# Combined effect of Tislelizumab and chemotherapy on tumor control rate and prognosis in patients with small cell lung cancer

**DOI:** 10.12669/pjms.41.5.11530

**Published:** 2025-05

**Authors:** Ning Zhong, Menglan Zhong, Wei Zhuang

**Affiliations:** 1 Ning Zhong, Department of Geriatric Oncology, Jiangxi Cancer Hospital & Institute, Jiangxi Clinical Research Center for Cancer, The Second Affiliated Hospital of Nanchang Medical College, 519 Beijing East Road, Nanchang, Jiangxi Province 330029, P.R. China; 2 Menglan Zhong, Department of Abdominal Surgical Oncology, Jiangxi Cancer Hospital & Institute, Jiangxi Clinical Research Center for Cancer, The Second Affiliated Hospital of Nanchang Medical College, 519 Beijing East Road, Nanchang, Jiangxi Province 330029, P.R. China; 3 Wei Zhuang, School of Nursing, Nanchang Medical College, 689 Huiren Ave, Nanchang City, Jiangxi Province, 330052, P.R. China

**Keywords:** Chemotherapy, Prognosis, Small cell lung cancer, Tislelizumab, Tumor control rate

## Abstract

**Objective:**

Exploring the effect of the combined regimen of Tislelizumab and chemotherapy on tumor control rate and prognosis in patients with small cell lung cancer (SCLC).

**Methods:**

This retrospective analysis included data of 80 SCLC patients treated in Jiangxi Cancer Hospital from April 2021 to April 2023. Forty patients who were treated with a combination of chemotherapy and Tislelizumab (the Combined group) were matched in a 1:1 ratio with a cohort receiving chemotherapy alone (the Chemotherapy group). Levels of tumor markers and T cell subsets in both groups were compared before and after treatment. After six months of follow-up, the early disease recurrence rate and survival rate of the two groups were compared.

**Results:**

The tumor control rate of the Combined group was higher than that of the Chemotherapy group (*P*<0.05). After treatment, the serum levels of carcinoembryonic antigen (CEA), neuron-specific enolase (NSE), and cytokeratin-19-fragment (CYFRA21-1) in both groups decreased compared to before treatment and were lower in the Combined group compared to the Chemotherapy group (*P*<0.05). Combined treatment was associated with higher levels of CD3^+^, CD4^+^, and CD4^+^/CD8^+^ (*P*<0.05). The disease recurrence rate was lower, and the survival rate was higher in the patients who received the combined treatment than those treated by the chemotherapy alone(*P*<0.05).

**Conclusions:**

Adopting the conventional combination of Tislelizumab and chemotherapy to treat SCLC can downregulate tumor marker levels, improve immune function, enhance treatment efficacy, and ensure disease prognosis. Longer follow-up is needed to confirm long-term prognostic benefits.

## INTRODUCTION

Small cell lung cancer (SCLC) is a high-grade neuroendocrine carcinoma, characterized by early widespread metastasis, rapid growth, high malignancy, and low differentiation, and accounts for 15%~20% of all lung cancer cases.^1^–^3^ Studies show that about 70% of SCLC patients are diagnosed in the extensive stage of the disease, with poor prognosis and a five years survival rate of less than 5%. Without active treatment, the average survival time of SCLC patients is only 2-4 months.^4^–^6^

Although platinum-based chemotherapy is currently considered a treatment of choice for SCLC, its overall effect is poor, with a high disease recurrence rate and only about 10% rate of disease-free survival after two years.^7^,^8^ Recently, numerous studies demonstrated the role of programmed cell death protein 1 (PD-1) and PD-1 ligand (PG-L1) in mediating cancer immune escape mechanisms, making this pathway a potential target of novel anti-tumor therapies.^9^,^10^ The research shows that an immune checkpoint inhibitor, Tislelizumab, a monoclonal antibody directed against PD-1, can reverse tumor-induced changes in the immune microenvironment, restore T cell proliferation and improve its functions, and strengthen the endogenous tumor immune effect.^11^,^12^

However, the research on the clinical application value of Tislelizumab in SCLC is scarce. This retrospective study aimed to clarify the therapeutic effect of combination therapy of Tislelizumab and conventional chemotherapy, focusing on disease recurrence and survival.

## METHODS

This retrospective analysis included clinical data of 80 SCLC patients treated in Jiangxi Cancer Hospital from April 2021 to April 2023. Based on gender, age, BMI, smoking status, alcohol consumption status, disease staging, and Eastern Cooperative Oncology Group (ECOG) performance status score, 40 patients who received standard chemotherapy with tislelizumab were matched 1:1 with a cohort receiving chemotherapy alone.

### Ethics Statement:

All procedures performed in the study involving human participants were by the ethical standards of the institutional and/or national research committee(s) and with the Helsinki Declaration (as revised in 2013). As this was a retrospective study and did not include any potentially identifiable patient data, informed consent was waived by the Ethics Committee of Jiangxi Cancer Hospital. The Medical Ethics Committee of Jiangxi Cancer Hospital approved this study (No.: 2021ky210, Date: September 28, 2021).

### Inclusion criteria:

- Meets the diagnostic criteria for SCLC.^1^
- SCLC diagnosed by cytology or histology.
- Age ≥ 18 years old.
- Receiving chemotherapy alone or combination therapy with Tislelizumab and chemotherapy.
- Complete clinical data (demographics, pathology, therapy, imaging, and follow-up).

### Exclusion criteria:

- Patients who have previously received PD-1 monoclonal antibody treatment.
- Patients with other malignant tumors.
- Patients with immune system disorders.
- Patients with substantial organ failure (defined as total bilirubin >1.5×, ALT/AST >2.5×, creatinine clearance <50 mL/min, or NYHA cardiac function classification ≥II)
- Patients with significant organ dysfunction.
- Patients with allergic constitution.
- Long-term use of immunosuppressive agents and hormone therapy.

### Treatment regimen:

The conventional etoposide and cisplatin (EP) chemotherapy regimen included intravenous infusion of etoposide (Kunyao Group Co., Ltd; 2ml:40mg) 100mg/m2 + cisplatin (Qilu Pharmaceutical Co., Ltd; 50mg:50ml) 30mg/m2 d1-3, three weeks per cycle, for a total of six cycles of treatment. Tislelizumab ((Baizean, Boehringer-Ingelheim Biopharmaceuticals (China) Co., Ltd; 10ml:100mg) was delivered intravenously (200 mg Tislelizumab) once every three weeks for a total of six sessions.

### Observation indicators:

- Disease control rate (DCR): According to response evaluation’s criteria in solid tumors (RECIST 1.1), the curative effect can be divided into complete response (CR), partial response (PR), stable disease (SD) and progressive disease (PD). Disease control rate = (CR+PR+SD)/total incidence× 100%.
- Serum levels of the tumor markers, including carcinoembryonic antigen (CEA), neuron-specific enolase (NSE), and cytokeratin-19-fragment (CYFRA21-1) were measured by enzyme-linked immunosorbent assay.
- Serum levels of T cell subpopulation indicators, including levels of CD3^+^, CD4^+^, CD4^+^/CD8^+^ were measured using a flow cytometer.
- Prognosis: disease recurrence and survival rate after six months of follow-up.

### Statistical Analysis:

All data analyses were conducted using SPSS 25.0 software (IBM Corp, Armonk, NY, USA). The measurement data were represented as mean ± standard deviation, an independent sample t-test was used for inter-group comparison, and a paired t-test was used for intra-group before and after comparison. The chi-square test was used to represent the number of cases. *P*<0.05 was considered statistically significant. The primary goal is disease control rate, while secondary end points include tumor marker levels, T cell index and survival outcomes. The calculation of sample size (α= 0.05, β = 0.2) shows that it takes 38 patients in each group to detect the disease control rate, which is expected to increase by 20.

## RESULTS

Clinical records of 80 patients (48 males and 32 females) were included in this study. The age of the patients ranged from 40 to 72 years, with an average of 54.47 ± 7.09 years. Based on the treatment received, 40 patients comprised the Chemotherapy group, and 40 were included in the Combined group. There was no significant inter-group difference in the baseline characteristics, including gender, age, body mass index (BMI), smoking status, alcohol consumption status), staging, and Eastern Cooperative Oncology Group (ECOG) score (*P*>0.05) (Table-I). The tumor control rate of the Combined group (87.50%) was higher than that of the Chemotherapy group (67.50%) (*P*<0.05) (Table-II).

**Table-I:** Comparison of General Information between Two Groups.

Item	Combined group (n=40)	Chemotherapy group (n=40)	t/χ^2^	P
Male (yes), n(%)	23 (57.50)	25 (62.50)	0.208	0.648
Age (year), mean ±SD	53.82±7.13	55.11±6.99	0.817	0.416
BMI (kg/m^2^), mean ±SD	22.67±3.44	23.05±3.08	0.520	0.604
Smoking (Yes), n(%)	21 (52.50)	24 (60.00)	0.457	0.499
Drinking alcohol (yes), n(%)	25 (62.50)	27 (67.50)	0.220	0.639
*Staging, n(%)*				
Extensive period	34 (85.00)	36 (90.00)	0.457	0.499
Limitation period	6 (15.00)	4 (10.00)		
*ECOG score, n(%)*				
1	22 (55.00)	24 (60.00)	0.205	0.651
2	18 (45.00)	16 (40.00)		

**Table-II:** Comparison of tumor control rates between two groups [n (%)].

Group	n	CP	PR	SD	PD	Tumor control rate
Combined group	40	6 (15.00)	25 (62.50)	4 (10.00)	5 (12.50)	35 (87.50)
Chemotherapy group	40	2 (5.00)	15 (37.50)	10 (25.00)	13 (32.50)	27 (67.50)
*χ^2^*						4.588
*P*						0.032

**Fig.1:**
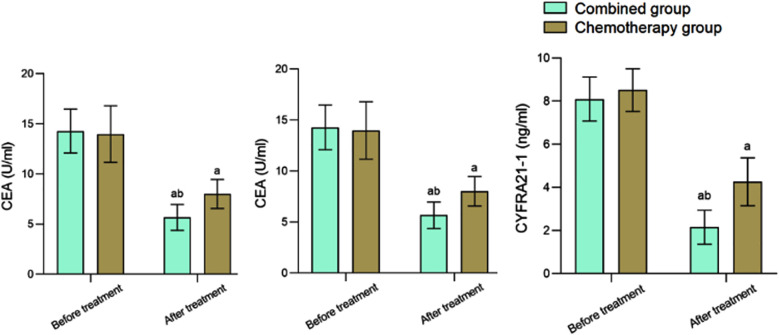
Comparison of tumor marker levels before and after treatment between two groups; Compared to before treatment in the same group. ^a^*P*<0.05; Compared to the Chemotherapy group, ^b^*P*<0.05; CEA: carcinoembryonic antigen NSE: neuron specific enolase; CYFRA21-1: Cytokeratin-19-fragment.

**Fig.2:**
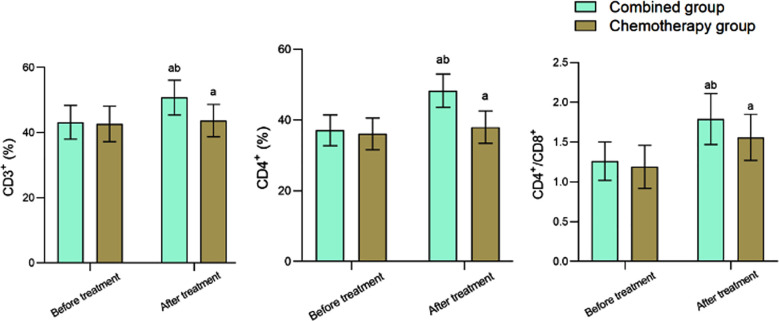
Comparison of T cell subsets index levels before and after treatment between two groups; Compared to before treatment in the same group. ^a^*P*<0.05; Compared to the Chemotherapy group, ^b^*P*<0.05.

Before the treatment, the two groups had no significant difference in serum CEA, NSE, and CYFRA21-1 levels (*P*>0.05). After the treatment, these indexes significantly decreased compared to the pre-treatment levels and were considerably lower in the Combined group compared to the Chemotherapy group (*P*<0.05) (Fig.1). Before the treatment, CD3+, CD4+, and CD4+/CD8+ levels were comparable in the two groups (*P*>0.05). After the treatment, CD3^+^, CD4^+^, and CD4^+^/CD8^+^ levels of the Combined group were significantly higher than those in the Chemotherapy group (*P*<0.05) (Fig.2). The disease recurrence rate in the Combined group was lower than that in the Chemotherapy group (12.50% versus 32.50%, respectively). The survival rate of the Combined group (85.00%) was considerably higher compared to the Chemotherapy group (65.00%) (*P*<0.05) (Table-III).

**Table-III:** Comparison of Prognosis between Two Groups [n (%)].

Group	n	Disease recurrence rate	Survival rate
Combined group	40	5 (12.50)	34 (85.00)
Chemotherapy group	40	13 (32.50)	26 (65.00)
*χ^2^*		4.588	4.267
*P*		0.032	0.039

## DISCUSSION

The results of this study showed the combined regimen of EP chemotherapy and Tislelizumab is more effective in downregulating tumor marker levels, improving immune function, and is associated with enhanced treatment efficacy and better disease prognosis compared to EP chemotherapy alone. Our results further confirm previous reports that using Tislelizumab in combination with conventional chemotherapy can reduce tumor marker levels, improve treatment efficacy, reduce disease recurrence, and prolong the survival of lung cancer patients.^13^–^15^ Zhou C et al.^16^ has showed that the median survival and overall survival in patients with advanced non-small cell lung cancer (NSCLC) who received Tislelizumab combined with docetaxel were 17.2 months and 11.9 months, respectively.

These results confirmed that Tislelizumab could help prolong the survival cycle of lung cancer patients, which is consistent with the viewpoint of this study. Liu SY et al.^17^ showed that the anti-tumor activity of Tislelizumab is stronger than traditional PD-1 monoclonal antibodies and is beneficial for improving tumor control rate and prolonging patient survival cycle. The research results of Huang D et al.^18^ showed that compared to conventional chemotherapy alone, the addition of Tislelizumab in the treatment of advanced lung cancer could effectively alleviate clinical symptoms and improve treatment effectiveness and the quality of life of patients. Tislelizumab can activate multiple signaling pathways in the body, contributing to an immunosuppressive tumor microenvironment state.^19^,^20^ The PD-1/PD-L1 signaling pathway can activate T cell proliferation and cytokine generation and exert an inhibitory effect that eventually allows cancer immune escape.^19^–^21^

A study by Lu S et al.^22^ that focused on the effect of combined Tislelizumab and chemotherapy as the first-line treatment of locally advanced/metastatic NSCLC showed that the combined treatment was associated with better response rates for tumor remission and superior median progression-free survival. A randomized controlled study by Messori et al.^23^ confirmed that chemotherapy combined with Tislelizumab for the first-line treatment of advanced lung cancer resulted in a median survival of 7.97 months. These results have a certain degree of consistency with the results of our study. These effects of Tislelizumab can be attributed to its activity as a monoclonal antibody that binds to PD-1 with high affinity and specificity and inhibits the binding of PD-1 to PD-L1, thus preventing T cell apoptosis, stimulating their cytokine activity, improving immune function and ultimately, inhibiting tumor cell growth.^13^,^22^–^24^

In agreement with this theory, this study demonstrated that CD3+, CD4+, and CD4+/CD8+ levels of patients treated using the combined regimen were higher than patients who received EP chemotherapy alone, consistent with previous research.^25^,^26^ Our results further confirm, that the combination of Tislelizumab and chemotherapy can improve the immune function of patients with SCLC. In summary, our report confirms that the combination of Tislelizumab and chemotherapy has outstanding clinical efficacy and safety in the treatment of SCLC patients in the short term. This has clinical significance for first-line treatment of SCLC. It is important to points out that we did not examine the relationship between these enhanced immunological markers and clinical outcomes (such as disease control rate, recurrence rate, and survival rate). This approach will be crucial in future research to ascertain whether the enhancement of T cell subsets can serve as a predictive biomarker for the therapeutic efficacy of tislelizumab.

### Limitations:

Firstly, this is a small sample size single-center retroactive study. Consequently, one should be careful to conclude that this mix treatment is better than chemotherapy by itself. Secondly, neither group was assigned at random. Baseline data could thus be biassed and unbalanced. Thirdly, more investigation on expanding and particular patient groups helps to validate the data of this study. Fourthly, based on the researcher’s assessment, patients who finished six months of treatment without documented illness progression were allowed to keep using tislelizumab and were regarded to still benefit from the trial medication. Another important restriction is that we did not methodically gather and document treatment-related adverse events, especially immune-related adverse events, which are absolutely vital for fully assessing the safety profile of tislelizumab used with chemotherapy. Clinical decision-making evaluating this treatment method depends on these safety data. At last, large-scale controlled studies including patients with additional etiologies should confirm the advantages of the combination therapy of tislelizumab with EP chemotherapy in SCLC patients.

## CONCLUSION

Combining conventional chemotherapy with Tislelizumab is more effective in downregulating tumor marker levels, improving the immune function of patients with SCLC. Combined regimen is associated with enhanced treatment efficacy, and better disease prognosis. Tillizumab plus chemotherapy is promising for SCLC treatment. Future large prospective studies need biomarker analysis to confirm these results and find the best medications.

### Authors’ contributions:

**NZ:** Study design, literature search, manuscript writing, revision, validation and Critical Analysis.

**MZ and WZ:** Data collection, data analysis and interpretation. Critical Review

All authors have read, approved the final manuscript and are responsible for the integrity of the study.
